# Legacy contaminants and low occurrence of PAHs in *Sotalia guianensis* from a highly impacted coastal region, Santos Basin, Brazil

**DOI:** 10.1007/s10661-026-15956-0

**Published:** 2026-09-25

**Authors:** Rafael Patrick R. Marcelino, Josilene da Silva, Raphael de Lucca Marcello Jarcovis, Cristian Taboada Timoszczuk, Felipe Rodrigues dos Santos, Ligia Dias de Araujo, Daniela Alves Maia da Silva, Satie Taniguchi, Rafael André Lourenço

**Affiliations:** 1 Oceanographic Institute , University of São Paulo, Praça do Oceanográfico, 191, Cidade Universitária, São Paulo, Brazil; 2 Oceanographic Institute , Federal University of Rio Grande, Rio Grande, Rio Grande do Sul Brazil

**Keywords:** Persistent organic pollutants, Polycyclic aromatic hydrocarbons, Marine mammals, Sotalia guianensis

## Abstract

Guiana dolphins (*Sotalia guianensis*) are coastal top predators exposed to persistent organic pollutants (POPs) and polycyclic aromatic hydrocarbons (PAHs). We evaluated PCBs, DDTs, and PAHs in liver tissues of 70 stranded individuals from the Santos Basin, southeastern Brazil. The maximum concentrations reached, in wet weight, 759 ng g⁻^1^ for total PCBs and 242 ng g⁻^1^ for total DDTs. Parent PAHs were quantified in 20 samples, predominantly as naphthalene, with concentrations up to 200 ng g⁻^1^. This indicates a low hepatic occurrence of the measured parent PAHs but does not necessarily imply low environmental exposure. Descriptively, PCB and DDT concentrations tended to be higher in individuals stranded in Rio de Janeiro and São Paulo than in Paraná and Santa Catarina, although interpretation is limited by the unbalanced sampling design and unknown movement histories. Fetuses generally showed lower PCB concentrations, while calves, juveniles, and adults exhibited substantial variability and overlapping concentration ranges, a pattern consistent with possible age-related accumulation but potentially influenced by other biological and geographic factors. Detection in fetal liver demonstrated prenatal exposure but did not allow maternal transfer to be quantified. Concentrations varied substantially among years between 2017 and 2022, but no consistent temporal trend was evident; annual sample sizes were small and uneven. Despite these limitations, this dataset provides an important regional baseline for future assessments of contaminant exposure in *Sotalia guianensis.*

**Supplementary Information:**

The online version contains supplementary material available at https://doi.org/10.1007/s10661-026-15956-0.

## Introduction

Coastal ecosystems are among the most impacted environments by anthropogenic activities, particularly in rapidly urbanized regions. In Brazil, intense coastal urbanization since the mid-twentieth century, driven by industrialization and rural-to-urban migration, has resulted in the widespread release of synthetic chemicals into the environment (Souza et al., 2024). These include pesticides, herbicides, lubricating oils, and flame retardants, which are frequently introduced into coastal systems during production, use, and disposal. As semi-enclosed and highly productive environments, coastal areas are especially vulnerable to contamination, with potential consequences for ecosystem health and human populations (Hardoim et al., 2021; Souza et al., 2024).

Among the contaminants of concern, persistent organic pollutants (POPs) and polycyclic aromatic hydrocarbons (PAHs) stand out due to their environmental persistence, toxicity, and capacity to accumulate in biota. POPs, including dichlorodiphenyltrichloroethanes (DDTs) and polychlorinated biphenyls (PCBs), are characterized by resistance to degradation associated with the stability of carbon–chlorine bonds (Jones & de Voogt, 1999), as well as high lipophilicity, which promotes bioaccumulation and biomagnification along trophic webs (Borgå et al., 2004; Tanabe, 1988). In addition, their semi-volatile nature enables long-range environmental transport (Ashraf, 2017), resulting in their widespread occurrence in sediments, water, and biota (Ashraf, 2017; Gorman et al., 2019; Montone et al., 2016; Mössner & Ballschmiter, 1997; Taniguchi et al., 2019).

Unlike POPs in some aspects, PAHs comprise a group of semi-volatile organic compounds formed primarily through incomplete combustion and petrogenic inputs (Neff, 1980). Many PAHs are toxic, with mutagenic and carcinogenic properties, and are therefore routinely included in environmental monitoring programs (USEPA, 1993, 1995a; Webster et al., 2018). Unlike POPs, PAHs are generally more susceptible to biotransformation, particularly in vertebrates, which limits their biomagnification potential (Lourenço et al., 2021; Nakata et al., 2003; Takeuchi et al., 2009; Wan et al., 2007). Nevertheless, continuous inputs and their persistence in sediments and suspended particles make PAHs environmentally relevant contaminants, especially in coastal regions influenced by urban and industrial activities.

Marine mammals, particularly coastal cetaceans, are highly susceptible to contamination by these compounds due to their ecological and physiological characteristics. As long-lived top predators with high lipid content and limited capacity to eliminate certain contaminants, these organisms integrate contaminant exposure over time and space. The Guiana dolphin (*Sotalia guianensis*), a coastal species widely distributed along the western Atlantic, is commonly found in estuaries and bays, where anthropogenic pressures are intense. This species is exposed to multiple stressors, including bycatch, habitat degradation, noise pollution, and chemical contamination (Laílson-Brito et al., 2010; Domit et al., 2023; Azevedo et al., 2009), and is currently classified as “Near Threatened” by the IUCN (Secchi et al., 2018).

The Santos Basin encompasses densely urbanized and industrialized coastal areas, major ports, estuaries, and offshore oil and gas activities. This combination of anthropogenic pressures makes the region particularly relevant for evaluating contaminant exposure in coastal cetaceans (Pereira et al., 2024; Souza et al., 2024). In southeastern Brazil, several studies have reported POP concentrations in marine organisms (Alonso et al., 2010; da Silva et al., 2003; Dorneles et al., 2013; Kajiwara et al., 2004; Lailson-Brito et al., 2010; Jarcovis et al., 2026; Quinete et al., 2011; Yogui et al., 2010). However, despite the ecological relevance of *Sotalia guianensis*, data on organic contaminants in this species remain limited. Most studies have focused on blubber, reflecting the lipophilic nature of POPs and the relative ease of sampling (De Wit, 2002), while other tissues, such as the liver, remain underexplored (Barbosa et al., 2018). Furthermore, information on PAH concentrations in marine mammals is scarce (Dron et al., 2023; Gui et al., 2018; Marsili, 2000), and no studies have reported PAHs for *Sotalia guianensis tissues*.

The liver plays a central role in the metabolism of xenobiotics in mammals, primarily through enzymatic systems such as cytochrome P450, which are responsible for the biotransformation of a wide range of compounds (Serafini et al., 2024). Therefore, liver tissue provides valuable insights into both contaminant exposure and metabolic processing, making it particularly relevant for integrated assessments of POPs and PAHs (Barbosa et al., 2018; Lourenço et al., 2023).

Given the contrasting environmental behavior of POPs and PAHs and the limited information available for *Sotalia guianensis*, especially in metabolically active tissues, there is a clear need to better understand the occurrence of these contaminants in this species. In this context, the present study aimed to evaluate the occurrence and concentrations of PCBs, DDTs, and PAHs in liver samples from *Sotalia guianensis* individuals stranded along the Santos Basin, providing baseline data on hepatic contaminant burdens in this coastal top predator.

## Methods

**Fig. 1:**
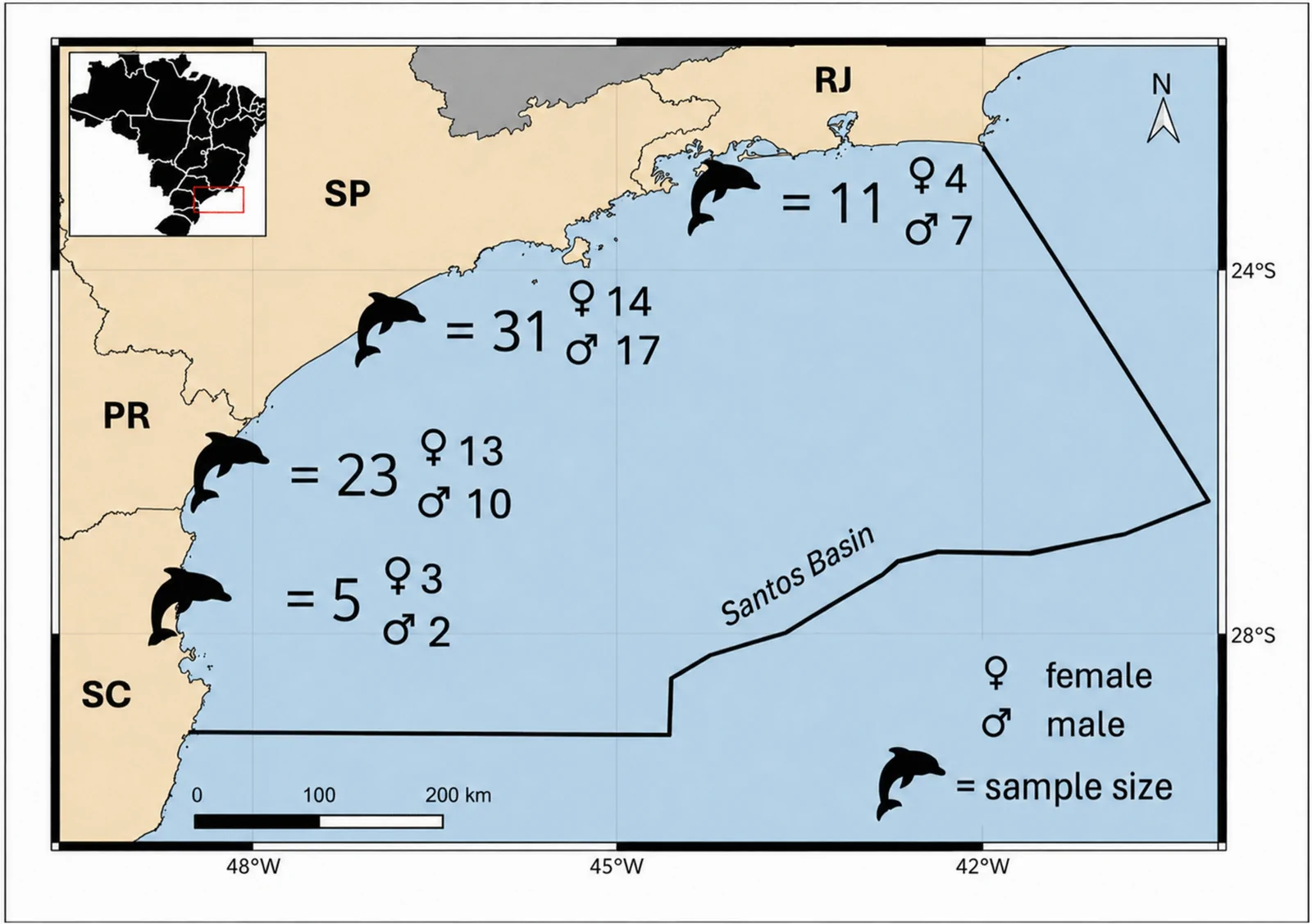
Sampling distribution of *Sotalia guianensis* along the Santos Basin, showing the total number of individuals (n) and their distribution by sex at each location along the Brazilian coast, from north to south: RJ (Rio de Janeiro), SP (São Paulo), PR (Paraná), and SC (Santa Catarina)

Liver samples of *Sotalia guianensis* (n = 70; 36 males and 34 females) were obtained from stranded individuals along the Santos Basin (Fig. 1), southeastern Brazil, between May 2017 and September 2022. Sampling covered the coasts of Rio de Janeiro (RJ, n = 11), São Paulo (SP, n = 31), Paraná (PR, n = 23), and Santa Catarina (SC, n = 5). Because sampling depended on the opportunistic recovery of stranded animals, sample allocation among states could not be predetermined or balanced. Consequently, geographic comparisons reflect the available stranded specimens, and results for Santa Catarina should be interpreted cautiously because of the small sample size.

The Santos Basin, the largest offshore sedimentary basin in Brazil, extends along the southeastern and southern Brazilian coast, from Cabo Frio to Florianópolis, and is currently subjected to intense oil and gas exploration activities (Silveira de Souza & Chaves-Sgarbi, 2019). The region encompasses diverse coastal and marine ecosystems, including estuaries, rocky shores, and continental shelf environments, which are subject to increasing anthropogenic pressures. Notably, the northern portion of the basin, particularly along the coasts of Rio de Janeiro and São Paulo states, is more industrialized than the southern portion, which may contribute to spatial differences in environmental contamination.

Necropsies were performed by veterinary teams following the same general procedures. Only carcasses classified as decomposition code 2 (fresh condition) were included (Geraci & Lounsbury, 1993). Sex was determined by examination of the external genital morphology and confirmed by gonadal inspection during necropsy. Individuals were classified as fetus, calf, juvenile, or adult according to body length and sexual maturity (Rosas & Monteiro-Filho, 2002). Fetuses were identified as individuals found in utero, calves as newborn or nursing animals, juveniles as sexually immature individuals, and adults as sexually mature animals based on gonadal development and species-specific length-at-maturity estimates (Rosas et al., 2003). Biological data, including sex, biometric measurements, and stranding location, were obtained from the SIMBA database (Petrobras, 2026).

Liver samples were collected during necropsies using pre-cleaned instruments, wrapped in aluminum foil, and stored at − 80 °C until analysis. Inner portions of the tissue were used to minimize external contamination. All procedures were conducted under appropriate ethical approvals (CEUA IO-USP, Protocol No. 004 Pesq. 2020) and collection permits (Abio No. 861/2017).

The analysis included polychlorinated biphenyls (PCBs; 26 congeners), dichlorodiphenyltrichloroethane and its metabolites (DDTs: DDT, DDD, and DDE isomers), and polycyclic aromatic hydrocarbons (PAHs). The latter comprised the 16 USEPA priority PAHs, selected alkylated homologs (Supplementary Material), and additional compounds such as dibenzothiophene, perylene, and benzo[e]pyrene. Alkylated PAHs and dibenzothiophenes were included as indicators of petrogenic inputs, whereas perylene and benzo[e]pyrene were included to complement the characterization of PAH profiles and potential sources.

Approximately 0.25 g of wet liver tissue was homogenized with anhydrous sodium sulfate and spiked with surrogate standards (PCB 103 for POPs and p-terphenyl-d14 for PAHs). Extraction was performed using Soxhlet with a mixture of n-hexane and dichloromethane (1:1, v/v) for 8 h, following the method USEPA 3540C.

Extracts were concentrated, and an aliquot was used for gravimetric lipid determination. The remaining extract was subjected to clean-up using silica/alumina column chromatography, following the methods USEPA 3610B and 3630C. Fractionation was performed by size-exclusion HPLC, and the fraction containing POPs and PAHs was collected, concentrated, and spiked with internal standards prior to analysis.

PCBs and PAHs were analyzed by gas chromatography coupled to mass spectrometry (GC–MS, Agilent 6890/5973N) operating in selected ion monitoring (SIM) mode, following USEPA 8270E. DDTs were determined using gas chromatography with electron capture detection (GC-ECD), following the method USEPA 8081B. Calibration curves were prepared using multi-level standards, showing good linearity (R^2^ > 0.99). Alkylated PAHs were quantified using the response factors of their corresponding parent compounds.

Moisture content was determined gravimetrically by drying subsamples to constant weight at 50 °C. Lipid content was determined from extract aliquots by solvent evaporation and gravimetric measurement. Where reported, lipid-normalized concentrations were calculated from wet-weight concentrations and the sample-specific lipid content according to: C_lw = C_ww/(lipid content/100), where C_lw and C_ww represent the lipid-weight and wet-weight concentrations, respectively.

Quality control procedures included the analysis of procedural blanks, spiked blanks, matrix spikes, and sample replicates within each batch. Surrogate recoveries were monitored for all samples.

Certified reference materials (NIST SRM 1945 and SRM 2974a) were analyzed periodically to assess accuracy. Limits of Detection (LOD) were calculated as three times the standard deviation of replicate analyses (Wade & Cantillo, 1994), and quantification limits were defined by the lowest calibration level.

No significant contamination was detected in blanks. Mean surrogate recoveries were 84.3 ± 9.7% for PCB 103 and 69.5 ± 9.8% for p-terphenyl-d14. Replicate analyses demonstrated good precision. Measured concentrations in reference materials were within certified ranges, indicating satisfactory accuracy and reliability of the analytical procedures based on USEPA (1995b).

Detailed information on extraction, cleanup, instrumental conditions, calibration, and analytical quality control is provided in the Supplementary Material.

### Data treatment and contaminant profile interpretation

ΣPCB, ΣDDT, and ΣPAH were calculated by summing only individual compounds with concentrations ≥ LOQ (USEPA, 2000); measurements below the LOQ were therefore excluded from these sums. PCB homolog contributions were expressed as percentages of ΣPCB, and the ΣDDT/ΣPCB ratio was used to describe the relative predominance of these contaminant classes, not as a definitive source-diagnostic indicator. PAH profiles were qualitatively evaluated based on molecular weight and the occurrence of parent compounds, alkylated homologs, and dibenzothiophenes. Diagnostic ratios were not calculated because of low detection frequency, limited compound co-occurrence, and potential alteration by hepatic metabolism.

Concentrations were expressed as ng g^−1^ wet weight and summarized using medians, ranges, detection or quantification frequencies, and graphical distributions. Detection was defined as a concentration ≥ LOD, whereas quantification was defined as a concentration ≥ LOQ; concentrations between the LOD and LOQ were considered detected but not quantified. For statistical summaries and boxplots, values below the LOD and LOQ were replaced by LOD/2 and LOQ/2 respectively. These substituted values were used exclusively for these purposes and were excluded from reported sums and ratios derived from them. For frequency calculations, the applicable criterion (detection or quantification) is specified in the corresponding figure or analysis. Log_10_ scaling was applied only to boxplots, and each individual was treated as an independent sampling unit. Given the opportunistic sampling of stranded animals, left-censored data, and small, strongly unequal groups, geographic, sex, developmental-stage, and temporal patterns were evaluated descriptively, without inferential comparisons, and should not be interpreted as statistically supported differences.

## Results and discussion

Considering all analyzed samples on a wet weight basis, total PCB concentrations, expressed as the sum of 26 congeners, ranged from not detected to 759 ng g^−1^. DDT concentrations, comprising p,p’-DDT, p,p’-DDE, p,p’-DDD, o,p’-DDT, o,p’-DDE, and o,p’-DDD, ranged from not detected to 242 ng g^−1^. The Supplementary Material presents individual data on PCBs, DDTs, and PAHs (wet weight), along with lipid and moisture contents. Additional information includes stranding location, body weight at necropsy, body condition score, sex, maturation/developmental stage, and sampling date for each individual.

To facilitate comparison with previous studies, concentrations were also expressed on a lipid-weight basis (lw). Lipid-normalized concentrations reached maximum values of 20,094 ng g^−1^ lw for ΣPCBs and 10,671 ng g^−1^ lw for ΣDDTs. These values should be interpreted cautiously because differences in tissue type, lipid content, target analytes, and analytical methods limit direct comparisons among studies.

Based on a review of the 10 studies reporting PCB and DDT concentrations in tissues of Sotalia spp. along the Brazilian coast (Table 1), concentrations reached up to 279,410 ng g^−1^ lipid weight for PCBs and 125,000 ng g^−1^ lipid weight for DDTs, both measured in blubber samples (Dorneles et al., 2013; Yogui et al., 2003). No studies reported DDT concentrations in liver tissues of Sotalia spp. For liver, only two studies reported PCB concentrations: a mean ∑51PCBs concentration of 790 ng g^−1^ wet weight (Quinete et al., 2011) and ∑51PCBs concentrations ranging from 1,520 to 26,300 ng g^−1^ lipid weight (Lavandier et al., 2015). Thus, the maximum lipid-normalized ΣPCB concentration observed in the present study (20,094 ng g^−1^ lw) falls within the range reported by Lavandier et al. (2015), whereas the mean wet-weight concentration reported by Quinete et al. (2011) is similar to and slightly higher than the maximum observed here (759 ng g^−1^ ww). These comparisons should be interpreted cautiously because of differences in summary statistics, analytical procedures, and reporting basis. Blubber primarily reflects long-term storage of lipophilic contaminants, whereas hepatic concentrations are more strongly influenced by redistribution and metabolic processing (Lourenço et al., 2021). Direct comparisons between liver and blubber concentrations should therefore be interpreted cautiously.

**Table 1:** Summary of studies reporting PCB and DDT concentrations in *Sotalia* spp. tissues along the Brazilian coast, SP: São Paulo, RJ: Rio de Janeiro, PR: Paraná, SC: Santa Catarina

Species	POPs levels	Tissue	Analytical method	Stranding location	Reference
*Sotalia guianensis*	μg g^-1^ wet weight / lipid weight Σ26PCB: <LOD-0.759 / <LOD-20.1 ΣDDT: <LOD-0.242 / <LOD-10.7	liver	Extraction: Soxhlet with dichloromethane-n-hexane (1:1, v:v) Clean-up: silica/alumina column and GPC Equipment: GC-ECD / GC-MS	Santos Basin (RJ, SP, PR, SC)	This work
*Sotalia fluviatilis*	μg g^-1^ lipid weight Σ27PCB: 0.2-9.22 ΣDDT: 0.54-125	blubber	Extraction: Soxhlet with dichloromethane-n-hexane (1:1, v:v) Clean-up: sulfuric acid treatment Equipment: GC-ECD	Cananéia Estuary, SP	Yogui et al., 2003
*Sotalia fluviatilis*	μg g^-1^ wet weight Σ6PCB: 2.62-8.99	blubber	Extraction: Soxhlet with n-hexane Clean-up: silica column Equipment: GC-ECD	Guanabara Bay, RJ	da Silva et al., 2003
*Sotalia guianensis*	μg g^-1^ lipid weight ΣPCB: 1.3-79 ΣDDT: 1-150	blubber	Extraction: Soxhlet with diethyl ether- n-hexane Clean-up: GPC and florisil column Equipment: GC-ECD	São Paulo and Paraná coasts, Brazil	Kajiwara et al., 2004
*Sotalia guianensis*	μg g^-1^ lipid weight Σ27PCB: 1.97 ΣDDT: 5.87	blubber	Extraction: Soxhlet with dichloromethane-n-hexane (1:1, v:v) Clean-up: sulfuric acid treatment Equipment: GC-ECD	São Paulo State Coast	Yogui et al., 2010
*Sotalia guianensis*	μg g^-1^ lipid weight Σ27PCB: 0.77-99.18 ΣDDT: 0.65-23.56	blubber	Extraction: Soxhlet with dichloromethane-n-hexane (1:1, v:v) Clean-up: sulfuric acid treatment Equipment: GC-ECD	Guanabara, Sepetiba,Ilha Grande, and Paranaguá bays, southeastern and southern Brazil	Lailson-Brito et al., 2010
*Sotalia guianensis*	μg g^-1^ lipid weight Σ31PCB: 25.87-66.03 ΣDDT: 16.91-55.91	blubber	Extraction: Tissumizer with dichloromethane-n-hexane (1:1, v:v) Clean-up: silica/alumina column and GPC Equipment: GC-ECD	São Paulo State Coast	Alonso et al., 2010
*Sotalia guianensis*	μg g^-1^ wet weight Mean Σ51PCB: 0.79	liver	Extraction: Ultra-turrax with acetone - n-hexane (1:1, v:v) Clean-up: saponification, alumina column, and sulfuric acid treatment Equipment: GC-MS	Paraíba do Sul river, RJ	Quinete et al., 2011
*Sotalia guianensis *	μg g^-1^ lipid weight Σ18PCB: 34.66 - 279.41	blubber	Extraction: ASE with n-hexane Clean-up: silica and alumina column Equipment: GC-MS	Rio de Janeiro State, Brazil	Dorneles et al., 2013
*Sotalia guianensis*	μg g^-1^ lipid weight Σ25PCB: 0.02-17.3 ΣDDT: 0.003-1.91	blubber	Extraction: Soxhlet with dichloromethane-n-hexane (1:1, v:v) Clean-up: sulfuric acid treatment Equipment: GC-ECD	Northeastern Brazil	Santos-Neto et al., 2014
*Sotalia guianensis*	μg g^-1^ lipid weight Σ51PCB: 1.52-26.3 Σ51PCB: 3.26-12.5	liver muscle	Extraction: Ultra-turrax with acetone, n-hexane and water after saponification. Clean-up: alumina column and sulfuric acid treatment Equipment: GC-MS	North-central coast of Rio de Janeiro State, Brazil	Lavandier et al., 2015

This review highlights considerable heterogeneity in the analytical protocols employed. The number of PCB congeners analyzed, for instance, ranges from 6 (da Silva et al., 2003) to 51 (Quinete et al., 2011), and this divergence relative to the 26 congeners analyzed in the present study limits direct comparability. In addition, variations are observed in extraction and clean-up procedures. Extraction techniques include Soxhlet extraction, accelerated solvent extraction (ASE), and tissumizer-based extraction, using solvents such as n-hexane, dichloromethane, diethyl ether, and acetone. Extract clean-up was performed using different approaches, including acidified silica columns, saponification, sulfuric acid treatment, and gel permeation chromatography.

In contrast, greater convergence is observed in the identification and quantification steps, which were predominantly performed using gas chromatography coupled to mass spectrometry (GC–MS) or electron capture detection (GC–ECD), generally with internal standardization. Analytical quality control procedures, however, are not consistently described, which may introduce additional uncertainty when comparing available data.

Furthermore, differences in data normalization hinder direct comparisons, as results are reported on both lipid weight and wet weight bases, in some cases without information on moisture and lipid content, preventing unit conversion. Another relevant factor is that most studies were conducted using samples from stranded animals, often lacking detailed information on carcass preservation status, which limits the assessment of compound integrity and the potential influence of external contamination.

**Fig. 2:**
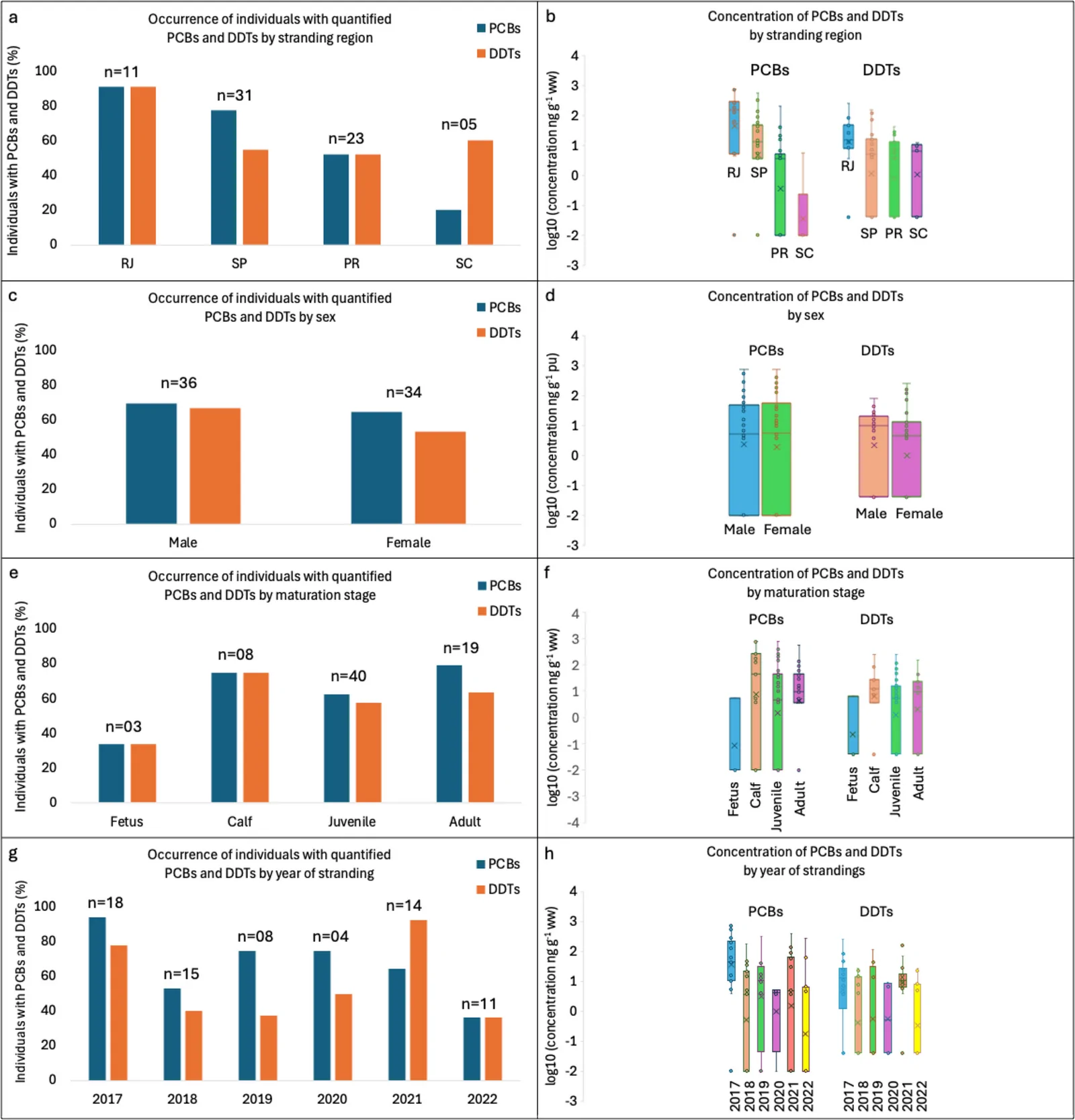
Percentage of individuals with at least one quantified PCB congener or DDT-related compound (left panels) and corresponding ΣPCB and ΣDDT concentrations (right panels) according to stranding location (a–b), sex (c–d), developmental stage (e–f), and year of stranding (g–h). Detection and quantification were defined as concentrations at or above the LOD and LOQ, respectively; values between the LOD and LOQ were considered detected but not quantified. For the boxplots, values below the LOD and LOQ were replaced by LOD/2 and LOQ/2 respectively, these substituted values were used only for graphical representation. Boxplots show the median, interquartile range, whiskers, and individual observations. Numbers above the bars indicate the total number of individuals in each group. RJ: Rio de Janeiro; SP: São Paulo; PR: Paraná; SC: Santa Catarina. Concentrations are expressed as ng g^−1^ wet weight on a log_10_ scale

Descriptively, PCB and DDT concentrations and quantification frequencies tended to be higher in individuals stranded in Rio de Janeiro and São Paulo than in those from Paraná and Santa Catarina (Fig. 2a, b). However, the small and unequal regional sample sizes, particularly for Santa Catarina, increase uncertainty and limit the interpretation of a north–south gradient. Stranding location was used only to group individuals geographically and should not be interpreted as the precise location of contaminant exposure. Although *Sotalia guianensis* generally exhibits site fidelity, the individual movement histories and home ranges of the sampled animals were unknown, and regional groups may also differ in age structure and nutritional or reproductive condition. Therefore, the observed concentrations cannot be unequivocally attributed to contaminant sources near the recovery locations.

Industrial and urban activities, including port operations, shipyards, petrochemical and metallurgical industries, electrical equipment handling, and the discharge of industrial and urban effluents, may have contributed to historical PCB inputs in Rio de Janeiro and São Paulo, particularly in highly impacted areas such as Guanabara Bay and the Santos estuarine system (da Silva et al., 2003). In contrast, higher relative contributions of DDTs in southern regions may reflect historical agricultural use and vector control programs.

The ΣDDT/ΣPCB ratio indicated a relative predominance of PCBs in Rio de Janeiro and São Paulo, where values were generally below one, and a greater relative contribution of DDTs in Paraná and Santa Catarina, where values were generally above one. Although this pattern may be compatible with differences in regional histories of industrialization and pesticide use (Jones & de Voogt, 1999; Meador et al., 1995), the ratio should not be interpreted as a definitive source-apportionment indicator. Tissue concentrations and their ratios may also be influenced by environmental persistence, dietary exposure, metabolism, age, sex, maternal transfer, movement history, and the treatment of non-detects. Although site fidelity reported for *Sotalia guianensis* may support some degree of local exposure (Azevedo et al., 2009; Santos et al., 2019), the absence of individual movement data precludes linking tissue concentrations directly to sources near the stranding locations.

PCB and DDT concentrations showed substantial overlap between sexes, with no clear differences in central tendency (Fig. 2c, d). However, quantification frequencies and concentrations were slightly higher in males, possibly associated with maternal offloading of contaminants during gestation and lactation (Aguilar et al., 1999; Barbosa et al., 2018).

PCB concentrations were generally lower in fetuses, whereas considerable variability and overlap were observed among calves, juveniles, and adults (Fig. 2e, f). This descriptive pattern is consistent with possible age-related accumulation but should not be interpreted as direct evidence of progressive bioaccumulation. Developmental stage may be associated with geographic origin, sex, body condition, lipid content, and sampling year, and the opportunistic and unbalanced sampling design did not allow these factors to be evaluated independently with sufficient statistical robustness.

Concentrations varied considerably within and among years between 2017 and 2022, but no consistent increasing or decreasing temporal tendency was apparent. The small and uneven annual sample sizes preclude stronger temporal inference.

Across PCB-positive samples, hexachlorinated congeners accounted, on average, for 88% of ΣPCB concentrations. This pattern is consistent with their higher persistence and bioaccumulation potential compared to lower chlorinated congeners (Beyer & Biziuk, 2009). The most abundant congeners were PCB 153, PCB 138, PCB 180, PCB 149, and PCB 101, in agreement with previous studies on *Sotalia guianensis* and other odontocetes (Lavandier et al., 2015; Quinete et al., 2011; Santos-Neto et al., 2014; Yogui et al., 2003). Their persistence is associated with structural features that hinder metabolic degradation (Kannan et al., 1995).

For DDTs, the distribution was strongly dominated by p,p’-DDE, detected in nearly all positive samples, while the parent compound (p,p’-DDT) was rarely observed. This pattern indicates predominantly aged contamination, reflecting environmental degradation and/or metabolic transformation of DDT (Zhang et al., 2018). Similar profiles have been reported in marine mammals worldwide (Carballo et al., 2008; Leonel et al., 2012).

PAHs were quantified in only 20 of the 70 samples, and in most positive samples (n = 15), only naphthalene was quantified. Total PAH concentrations ranged from < LOD to 200 ng g⁻^1^ ww, with a maximum of 6880 ng g⁻^1^ on a lipid-weight basis. This low quantification frequency indicates a low hepatic occurrence of the measured parent PAHs but does not necessarily imply low environmental exposure. Parent PAH concentrations in liver may be influenced by tissue distribution and rapid metabolism (Lourenço et al., 2021; Nakata et al., 2003; Takeuchi et al., 2009; Wan et al., 2007), although biotransformation was not directly evaluated in the present study.

The non-detection of alkylated PAHs and dibenzothiophenes does not exclude petrogenic exposure because these compounds may have been metabolized or occurred below the detection limits (Lourenço et al., 2021, 2023). Therefore, the available data do not allow definitive conclusions regarding PAH sources or exposure intensity. Measurements of PAH metabolites, cytochrome P450 activity, or other biomarkers would be required to evaluate exposure and biotransformation more directly (Neff, 2002; Varanasi et al., 1989).

PCBs and DDTs were detected more frequently than parent PAHs, consistent with their greater persistence and tissue retention. Nevertheless, differences in detection frequency among contaminant classes should not be interpreted as direct differences in environmental exposure intensity.

Tissue concentrations reflect the combined influence of dietary exposure, movement patterns, metabolism, age-related accumulation, and historical contamination, and should therefore not be interpreted as direct measures of local environmental concentrations. Because this study relied on stranded animals, the dataset may also be influenced by health condition and may not fully represent the living population. Hepatic PAH concentrations should be interpreted cautiously because rapid metabolism may reduce parent-compound concentrations, while comparisons with studies based on blubber or other tissues are limited by tissue-specific accumulation and metabolic processes. Despite these limitations, this regional dataset provides an important baseline for future assessments of spatial and temporal changes in contaminant exposure.

Toxicological thresholds established specifically for the liver of *Sotalia guianensis* are currently unavailable, and thresholds derived from blubber or other species cannot be directly applied. Consequently, biological effects cannot be inferred from tissue concentrations alone. Nevertheless, establishing regional baseline values is particularly relevant for a near threatened coastal species exposed to multiple anthropogenic pressures.

## Conclusion

This study documents the occurrence of PCBs and DDTs in liver tissues of *Sotalia guianensis* stranded along the Santos Basin and provides the first information on hepatic parent PAHs for this species. PCBs were detected more frequently and generally predominated over DDTs in the analyzed samples.

Descriptively, PCB and DDT concentrations tended to be higher in individuals stranded in Rio de Janeiro and São Paulo than in those from Paraná and Santa Catarina. The ΣDDT/ΣPCB ratios indicated a greater relative predominance of PCBs in the northern portion of the study area and of DDTs in the southern portion. However, the unbalanced sample sizes, unknown individual movement histories, and potential influence of biological factors prevent these patterns from being attributed unequivocally to sources near the stranding locations. PCB levels tended to be lower in fetuses, whereas concentrations among calves, juveniles, and adults were highly variable and broadly overlapping, a pattern consistent with possible age-related accumulation but not direct evidence of progressive bioaccumulation. Detection in fetal liver demonstrated prenatal exposure, although maternal transfer could not be quantified. Concentrations varied among years, but no consistent temporal trend was evident between 2017 and 2022. Parent PAHs occurred infrequently and predominantly as naphthalene. Their low hepatic occurrence should not be interpreted as evidence of low environmental exposure or limited accumulation because PAH metabolites and biomarkers of biotransformation were not evaluated. Overall, despite the limitations inherent to opportunistic sampling of stranded animals, this dataset establishes an important regional baseline for future assessments of spatial and temporal variation in contaminant exposure in this Near Threatened coastal species.

## Supplementary Information

Below is the link to the electronic supplementary material.

Supplementary file1 (XLSX 344 KB)

Supplementary file2 (DOCX 61.1 KB)

## Data Availability

All data supporting the findings of this study are available within the paper and its Supplementary Information.
