# Supplementary material for: Legacy contaminants and low occurrence of PAHs in Sotalia guianensis from a highly impacted coastal region, Santos Basin, Brazil
Source: Environ Monit Assess. 2026 Sep 25;198(10):1114. doi: 10.1007/s10661-026-15956-0 (PMC13612654; doi:10.1007/s10661-026-15956-0)
Supplement: Supplementary file 1 [file 10661_2026_15956_MOESM2_ESM.docx]

**Supplementary material for**

**Legacy contaminants and low occurrence of PAHs in *Sotalia guianensis* from a highly impacted coastal region, Santos Basin, Brazil.**

Rafael Patrick R. Marcelino**^1^**^*^, Josilene da Silva**^1^**, Raphael de Lucca Marcello Jarcovis**^1^**, Cristian Taboada Timoszczuk**^1^**, Felipe Rodrigues dos Santos**^1^**, Ligia Dias de Araujo**^1,2^**, Daniela Alves Maia da Silva**^1^**, Satie Taniguchi**^1^**, Rafael André Lourenço**^1*^**

**^1^** Oceanographic Institute – University of São Paulo, Brazil. Praça do Oceanográfico, 191, Cidade Universitária, **São Paulo**, São Paulo, Brazil

**^2^** Oceanographic Institute - Federal University of Rio Grande, **Rio Grande**, Rio Grande do Sul, Brazil

Author's email address:

* Rafael Patrick R. Marcelino <rafaelpatrick@usp.br>

Josilene Silva <josilenehsilva@gmail.com>

Rafael de Lucca Marcello Jarcovis <raphaeljarcovis94@gmail.com>

Cristian Taboada Timoszczuk <cristianzcz@gmail.com>

Felipe Rodrigues Santos <feliperod096@gmail.com>

Ligia Dias Araujo <ligia.araujo@furg.br>

Daniela Alves Maia da Silva <daniela.alvesmaia@gmail.com>

Satie Taniguchi <satie@usp.br>

*Rafael Andre Lourenço <rafaell@usp.br

**Detailed Analytical Procedure**

In this study, the 16 priority polycyclic aromatic hydrocarbons (PAHs) established by the United States Environmental Protection Agency (USEPA) were analyzed (naphthalene, acenaphthylene, acenaphthene, fluorene, phenanthrene, anthracene, fluoranthene, pyrene, benz[a]anthracene, chrysene, benzo[b]fluoranthene, benzo[k]fluoranthene, benzo[a]pyrene, indeno[1,2,3-c,d]pyrene, dibenz[a,h]anthracene, and benzo[ghi]perylene), along with dibenzothiophene, perylene, and benzo[e]pyrene. In addition, the following alkylated PAHs were considered: C1–C4 naphthalenes, C1–C3 fluorenes, C1–C3 dibenzothiophenes, C1–C4 phenanthrenes–anthracenes, C1–C2 fluoranthenes–pyrenes, and C1–C2 chrysenes.

For organochlorine compounds of the polychlorinated biphenyl (PCB) class, the following congeners were analyzed: PCB 49, 52, 66, 77, 81, 95, 101, 110, 114, 118, 123, 138, 141, 149, 151, 153, 156, 157, 169, 174, 177, 180, 189, 194, 195, and 206.

For organochlorine pesticides, the following compounds were analyzed: hexachlorobenzene (HCB); hexachlorocyclohexanes (α-, β-, δ-, and γ-HCH); DRINs (aldrin, isodrin, dieldrin, and endrin); chlordanes (heptachlor, heptachlor epoxide A and B, oxychlordane, α- and γ-chlordane); dichlorodiphenyls (o,p’-DDE, p,p’-DDE, o,p’-DDD, p,p’-DDD, o,p’-DDT, p,p’-DDT); endosulfan I and II; methoxychlor; and mirex.

For organobrominated compounds, the following polybrominated diphenyl ethers (PBDEs) were analyzed: PBDE 28, 47, 99, 100, 153, 154, and 183. Only PCBs and DDTs were considered for this work.

Analytical procedures followed USEPA methods 3540C (extraction), 3610, 3630, and 3640 (cleanup), and 8270E and 8081B (quantification), with adaptations based on NOAA protocols (Wade and Cantillo, 1994) and recent literature.

**Materials**

All materials (glassware, tweezers, blades, etc.) were rinsed with running water and washed in an automatic system (Miele GG05, Germany, model PG8583CD) at 60 °C using an alkaline detergent (22 HPF-x), followed by a neutralizing detergent (25-Organacid). Materials were dried using heated air in the same system.

After drying, all materials were decontaminated with dichloromethane and n-hexane prior to use. Alternatively, when appropriate, materials were combusted at 400 °C for 4 hours.

Drying agents and adsorbents such as sodium sulfate (J.T. Baker, USA), silica, and alumina (Merck, Germany) were combusted at 400 °C before use. Solvents (n-hexane and dichloromethane, J.T. Baker) were pesticide-grade. Water used to deactivate silica and alumina was deionized and subsequently extracted three times with n-hexane.

**Moisture Content Determination**

Moisture content was determined gravimetrically. Approximately 0.3–0.5 g of each tissue sample was transferred to a pre-weighed glass vial and kept in an open oven at 50 °C until constant weight was achieved. Moisture content (%) was calculated from the difference between wet and dry mass.

**Extraction**

Extraction of organism tissues followed USEPA method 3540C. Approximately 0.25 g of previously homogenized wet tissue was macerated with 10 g of anhydrous sodium sulfate.

Surrogate standards were added: p-terphenyl-d14 (500 ng) for PAHs and PCB 103 (100 ng) for POPs. Extraction was performed in a Soxhlet system for 8 hours using n-hexane and dichloromethane (1:1, v:v).

Extracts were concentrated to 1 mL using a rotary evaporator under reduced pressure or a TurboVap® II system (Biotage), and a 0.1 mL aliquot was removed for lipid determination.

**Lipid Content Determination**

An aliquot of 0.1 mL of the concentrated extract was transferred to a pre-weighed glass vial. After complete solvent evaporation at room temperature, the vial was reweighed, and lipid content (%) was calculated from the residue mass.

**Cleanup**

Cleanup followed adapted USEPA methods 3610, 3630, and 3640. The remaining 0.9 mL of each extract was eluted with 80 mL of n-hexane:dichloromethane (1:1, v:v) through a glass column packed with silica gel, alumina, and sodium sulfate.

Silica and alumina were previously deactivated with 5% water (deionized and n-hexane-extracted). The eluate was concentrated to 1 mL and injected into an HPLC system (Agilent 1260 Infinity II) equipped with a UV detector (254 nm) for fraction collection.

The 0–30 min fraction was discarded, and the 30–40 min fraction containing PAHs and POPs was collected. Internal standards were then added: deuterated PAHs (500 ng mL⁻¹ each) and TCMX (100 ng mL⁻¹), yielding a final volume of 900 µL.

**Quantification**

PAHs were analyzed by gas chromatography–mass spectrometry (GC-MS, Agilent 6890/5973N) operating in selected ion monitoring (SIM) mode, following USEPA 8270E.

Calibration curves ranged from 2.5 to 500 ng mL⁻¹, with R² > 0.99. Alkylated PAHs were quantified using calibration curves of their corresponding parent compounds.

PCBs and PBDEs were also analyzed by GC-MS (SIM mode), while organochlorine pesticides were analyzed by gas chromatography with electron capture detection (GC-ECD), following USEPA 8081B.

All calibration curves showed coefficients of determination (R²) greater than 0.99. Quantification was based on internal standardization.

Limits of detection (LOD) were defined as three times the standard deviation of seven replicates (MDL = 3 × SD), and limits of quantification (LOQ) corresponded to the lowest calibration point.

**Quality Control**

Quality control procedures included the analysis of a procedural blank (sodium sulfate), a spiked blank, and a spiked sample with each batch of approximately 20 samples. Spiking levels were 50 ng g⁻¹ for PAHs and 10 ng g⁻¹ for pesticides, PCBs, and PBDEs (wet weight).

Certified reference materials were also analyzed: NIST SRM 2974a (freeze-dried mussel tissue) for PAHs and SRM 1945 (whale blubber) for pesticides, PCBs, and PBDEs.

Results were considered valid when surrogate recoveries and spike recoveries ranged between 45% and 120%. Samples outside this range were reanalyzed. Analyses of reference materials confirmed the accuracy and precision of the analytical method.

**Table S1**. Results for blanks, fortified blanks, and fortified matrix samples for pesticides, PCBs, PBDEs and PAHs in this study (ng g^-1^), including percentage recovery. Acceptable recovery range: 45 to 120%.

|  | **Blank** | **Expected**  **Concent.** | **Spiked blank** | **Expected**  **Concent.** | **Recovery (%)** | **Spiked sample** | **Expected**  **Concent.** | **Recovery (%)** |
| --- | --- | --- | --- | --- | --- | --- | --- | --- |
|  |  |  |  |  |  |  |  |  |
| **Pesticides** |  |  |  |  |  |  |  |  |
| HCB | <0.08 | <0.08 | 6.65 | 10.00 | 66 | 6.85 | 10.00 | 69 |
| α-HCH | <0.05 | <0.05 | 7.09 | 10.00 | 71 | 6.74 | 10.00 | 67 |
| β-HCH | <0.13 | <0.13 | 6.06 | 10.00 | 61 | 6.07 | 10.00 | 61 |
| γ-HCH | <0.38 | <0.38 | 7.04 | 10.00 | 70 | 7.16 | 10.00 | 72 |
| δ-HCH | <0.02 | <0.02 | 6.23 | 10.00 | 62 | 7.35 | 10.00 | 73 |
| Aldrin | <0.09 | <0.09 | 7.03 | 10.00 | 70 | 7.01 | 10.00 | 70 |
| Isodrin | <0.09 | <0.09 | 6.51 | 10.00 | 65 | 7.48 | 10.00 | 75 |
| Dieldrin | <0.07 | <0.07 | 6.69 | 10.00 | 67 | 7.13 | 10.00 | 71 |
| Endrin | <0.10 | <0.10 | 6.79 | 10.00 | 68 | 6.62 | 10.00 | 66 |
| Heptachlor | <0.09 | <0.09 | 6.93 | 10.00 | 69 | 7.41 | 10.00 | 74 |
| Heptachlor epoxide A | <0.09 | <0.09 | 7.48 | 10.00 | 75 | 6.08 | 10.00 | 61 |
| Heptachlor epoxide B | <0.09 | <0.09 | 6.10 | 10.00 | 61 | 6.93 | 10.00 | 69 |
| Oxychlordane | <0.13 | <0.13 | 7.50 | 10.00 | 75 | 7.34 | 10.00 | 73 |
| γ-chlordane | <0.09 | <0.09 | 7.30 | 10.00 | 73 | 6.76 | 10.00 | 68 |
| α-chlordane | <0.17 | <0.17 | 7.18 | 10.00 | 72 | 6.31 | 10.00 | 63 |
| o,p'-DDE | <0.08 | <0.08 | 6.17 | 10.00 | 62 | 7.04 | 10.00 | 70 |
| p,p'-DDE | <0.08 | <0.08 | 6.27 | 10.00 | 63 | 7.34 | 10.00 | 73 |
| o,p'-DDD | <0.08 | <0.08 | 7.29 | 10.00 | 73 | 7.30 | 10.00 | 73 |
| p,p'-DDD | <0.08 | <0.08 | 7.17 | 10.00 | 72 | 6.54 | 10.00 | 65 |
| o,p'-DDT | <0.08 | <0.08 | 6.49 | 10.00 | 65 | 6.13 | 10.00 | 61 |
| p,p'-DDT | <0.08 | <0.08 | 6.31 | 10.00 | 63 | 6.66 | 10.00 | 67 |
| Endosulfan I | <0.04 | <0.04 | 7.10 | 10.00 | 71 | 6.35 | 10.00 | 63 |
| Endosulfan II | <0.05 | <0.05 | 6.33 | 10.00 | 63 | 6.49 | 10.00 | 65 |
| Metoxychlor | <0.05 | <0.05 | 6.48 | 10.00 | 65 | 6.50 | 10.00 | 65 |
| Mirex | <0.04 | <0.04 | 6.47 | 10.00 | 65 | 7.16 | 10.00 | 72 |
|  |  |  |  |  |  |  |  |  |
| **PCBs** |  |  |  |  |  |  |  |  |
| PCB 49 | <0.09 | <0.09 | 6.31 | 10.00 | 63 | 6.15 | 10.00 | 62 |
| PCB 52 | <0.09 | <0.09 | 6.56 | 10.00 | 66 | 6.04 | 10.00 | 60 |
| PCB 66 | <0.09 | <0.09 | 6.01 | 10.00 | 60 | 7.39 | 10.00 | 74 |
| PCB 77 | <0.09 | <0.09 | 7.48 | 10.00 | 75 | 6.22 | 10.00 | 62 |
| PCB 81 | <0.07 | <0.07 | 6.88 | 10.00 | 69 | 6.62 | 10.00 | 66 |
| PCB 95 | <0.06 | <0.06 | 6.67 | 10.00 | 67 | 7.07 | 10.00 | 71 |
| PCB 101 | <0.07 | <0.07 | 6.44 | 10.00 | 64 | 6.72 | 10.00 | 67 |
| PCB 110 | <0.06 | <0.06 | 6.67 | 10.00 | 67 | 6.36 | 10.00 | 64 |
| PCB 114 | <0.09 | <0.09 | 6.25 | 10.00 | 62 | 6.58 | 10.00 | 66 |
| PCB 118 | <0.07 | <0.07 | 7.09 | 10.00 | 71 | 6.17 | 10.00 | 62 |
| PCB 123 | <0.08 | <0.08 | 7.30 | 10.00 | 73 | 7.19 | 10.00 | 72 |
| PCB 138 | <0.05 | <0.05 | 6.41 | 10.00 | 64 | 6.31 | 10.00 | 63 |
| PCB 141 | <0.05 | <0.05 | 7.42 | 10.00 | 74 | 6.95 | 10.00 | 70 |
| PCB 149 | <0.05 | <0.05 | 6.72 | 10.00 | 67 | 7.49 | 10.00 | 75 |
| PCB 151 | <0.05 | <0.05 | 7.39 | 10.00 | 74 | 7.09 | 10.00 | 71 |
| PCB 153 | <0.12 | <0.12 | 7.35 | 10.00 | 74 | 6.03 | 10.00 | 60 |
| PCB 156 | <0.10 | <0.10 | 6.07 | 10.00 | 61 | 7.34 | 10.00 | 73 |
| PCB 157 | <0.06 | <0.06 | 6.10 | 10.00 | 61 | 6.67 | 10.00 | 67 |
| PCB 169 | <0.09 | <0.09 | 7.40 | 10.00 | 74 | 6.81 | 10.00 | 68 |
| PCB 174 | <0.06 | <0.06 | 7.09 | 10.00 | 71 | 7.09 | 10.00 | 71 |
| PCB 177 | <0.05 | <0.05 | 7.10 | 10.00 | 71 | 7.10 | 10.00 | 71 |
| PCB 180 | <0.05 | <0.05 | 6.75 | 10.00 | 68 | 6.36 | 10.00 | 64 |
| PCB 189 | <0.08 | <0.08 | 7.12 | 10.00 | 71 | 7.23 | 10.00 | 72 |
| PCB 194 | <0.07 | <0.07 | 6.82 | 10.00 | 68 | 7.10 | 10.00 | 71 |
| PCB 195 | <0.05 | <0.05 | 6.78 | 10.00 | 68 | 7.33 | 10.00 | 73 |
| PCB 206 | <0.02 | <0.02 | 7.46 | 10.00 | 75 | 7.33 | 10.00 | 73 |
|  |  |  |  |  |  |  |  |  |
| **PBDEs** |  |  |  |  |  |  |  |  |
| PBDE 28 | <0.05 | <0.05 | 7.50 | 10.00 | 75 | 7.40 | 10.00 | 74 |
| PBDE 47 | <0.05 | <0.05 | 7.07 | 10.00 | 71 | 7.49 | 10.00 | 75 |
| PBDE 99 | <0.05 | <0.05 | 7.03 | 10.00 | 70 | 6.70 | 10.00 | 67 |
| PBDE 100 | <0.10 | <0.10 | 6.28 | 10.00 | 63 | 6.14 | 10.00 | 61 |
| PBDE 153 | <0.07 | <0.07 | 6.76 | 10.00 | 68 | 6.56 | 10.00 | 66 |
| PBDE 154 | <0.06 | <0.06 | 7.37 | 10.00 | 74 | 7.46 | 10.00 | 75 |
| PBDE 183 | <0.09 | <0.09 | 6.55 | 10.00 | 66 | 6.89 | 10.00 | 69 |
|  |  |  |  |  |  |  |  |  |
| **PAHs** |  |  |  |  |  |  |  |  |
| Naphthalene | <0.6 | <0.6 | 37.8 | 50.0 | 76 | 31.8 | 50.0 | 64 |
| Acenaphthylene | <0.1 | <0.1 | 47.6 | 50.0 | 95 | 42.7 | 50.0 | 85 |
| Acenaphthene | <0.1 | <0.1 | 37.7 | 50.0 | 75 | 39.6 | 50.0 | 79 |
| Fluorene | <0.1 | <0.1 | 37.5 | 50.0 | 75 | 39.2 | 50.0 | 78 |
| Dibenzothiophene | <0.2 | <0.2 | 39.3 | 50.0 | 79 | 41.8 | 50.0 | 84 |
| Phenanthrene | <0.3 | <0.3 | 42.5 | 50.0 | 85 | 46.5 | 50.0 | 93 |
| Anthracene | <0.1 | <0.1 | 39.6 | 50.0 | 79 | 44.4 | 50.0 | 89 |
| Fluoranthene | <0.2 | <0.2 | 38.6 | 50.0 | 77 | 39.8 | 50.0 | 80 |
| Pyrene | <0.1 | <0.1 | 39.5 | 50.0 | 79 | 39.4 | 50.0 | 79 |
| Benz[*a*]anthracene | <0.3 | <0.3 | 42.9 | 50.0 | 86 | 45.9 | 50.0 | 92 |
| Chrysene | <0.3 | <0.3 | 34.0 | 50.0 | 68 | 36.9 | 50.0 | 74 |
| Benzo[*b*]fluoranthene | <0.2 | <0.2 | 43.2 | 50.0 | 86 | 46.4 | 50.0 | 93 |
| Benzo[*k*]fluoranthene | <0.2 | <0.2 | 32.5 | 50.0 | 65 | 42.0 | 50.0 | 84 |
| Benzo[*e*]pyrene | <0.1 | <0.1 | 36.8 | 50.0 | 74 | 47.4 | 50.0 | 95 |
| Benzo[*a*]pyrene | <0.1 | <0.1 | 34.0 | 50.0 | 68 | 46.8 | 50.0 | 94 |
| Perylene | <0.1 | <0.1 | 32.5 | 50.0 | 65 | 40.0 | 50.0 | 80 |
| Indeno[1,2,3-*c*,*d*]pyrene | <0.1 | <0.1 | 37.4 | 50.0 | 75 | 40.5 | 50.0 | 81 |
| Dibenz[*a*,*h*]anthracene | <0.1 | <0.1 | 45.3 | 50.0 | 91 | 43.8 | 50.0 | 88 |
| Benzo[*g*,*h*,*i*]perylene | <0.1 | <0.1 | 46.8 | 50.0 | 94 | 35.8 | 50.0 | 72 |

**Table S2**. Expected and measured concentrations for the certified reference materials (SRM 1945 for pesticides, PCBs, DDTs, and PBDEs, and SRM 2974a for PAHs) (ng g^-1^). Compounds not included in the certified reference materials are reported as not available (—).

|  | **Measured**  **Concent.** | **Expected**  **Concent.** |
| --- | --- | --- |
|  |  |  |
| **Pesticides** |  |  |
| HCB | 28.6 | 30.6 ± 1.5 |
| α-HCH | 15 | 16.9 ± 1.4 |
| β-HCH | — | — |
| γ-HCH | 2.99 | 3.18 ± 0.01 |
| δ-HCH | — | — |
| Aldrin | — | — |
| Isodrin | — | — |
| Dieldrin | 46.3 | 50.1 ± 4.1 |
| Endrin | — | — |
| Heptachlor | — | — |
| Heptachlor epoxide A | — | — |
| Heptachlor epoxide B | — | — |
| Oxychlordane | 20.1 | 21.2 ± 1.1 |
| γ-chlordane | 10.8 | 11.8 ± 0.5 |
| α-chlordane | 47 | 48.1 ± 1.6 |
| o,p'-DDE | 14.9 | 14.2 ± 1.4 |
| p,p'-DDE | 480 | 497 ± 19 |
| o,p'-DDD | 18.5 | 19.5 ± 1.2 |
| p,p'-DDD | 112 | 120 ± 5 |
| o,p'-DDT | 84 | 91 ± 14 |
| p,p'-DDT | 225 | 233 ± 8 |
| Endosulfan I | — | — |
| Endosulfan II | — | — |
| Metoxychlor | — | — |
| Mirex | 29.5 | 31.0 ± 3.4 |
|  |  |  |
| **PCBs** |  |  |
| PCB 49 | 18.0 | 18.3 ± 0.1 |
| PCB 52 | 39.6 | 40.7 ± 1.3 |
| PCB 66 | 22.0 | 22.4 ± 0.5 |
| PCB 77 | — | — |
| PCB 81 | — | — |
| PCB 95 | 33.1 | 33.9 ± 0.5 |
| PCB 101 | 69.1 | 78.0 ± 12.0 |
| PCB 110 | 31.2 | 33.8 ± 2.3 |
| PCB 114 | — | — |
| PCB 118 | 74.1 | 76.5 ± 2.9 |
| PCB 123 | — | — |
| PCB 138 | 152.0 | 146 ± 13 |
| PCB 141 | — | — |
| PCB 149 | 83.6 | 89.0 ± 6.9 |
| PCB 151 | 27.1 | 28.6 ± 1.3 |
| PCB 153 | 210.0 | 228 ± 10 |
| PCB 156 | 10.9 | 11.4 ± 0.9 |
| PCB 157 | — | — |
| PCB 169 | — | — |
| PCB 174 | 24.0 | 25.2 ± 0.1 |
| PCB 180 | 129.0 | 138 ± 10 |
| PCB 189 | — | — |
| PCB 194 | 49.2 | 53.5 ± 5.2 |
| PCB 195 | 12.2 | 14.3 ± 2.3 |
| PCB 206 | 40.0 | 44.9 ± 4.2 |
|  |  |  |
| **PBDEs** |  |  |
| PBDE 28 | — | — |
| PBDE 47 | 39.3 | 39.6 ± 0.2 |
| PBDE 99 | 17.9 | 18.9 ± 2.3 |
| PBDE 100 | 9.9 | 10.3 ± 1.1 |
| PBDE 153 | 8.1 | 8.34 ± 0.55 |
| PBDE 154 | 12.2 | 13.3 ± 1.7 |
| PBDE 183 | — | — |
|  |  |  |
| **PAHs** |  |  |
| Naphthalene | 9.56 | 9.68 ± 0.67 |
| Acenaphthylene | — | — |
| Acenaphthene | — | — |
| Fluorene | — | — |
| Dibenzothiophene | — | — |
| Phenanthrene | 73.6 | 74.4 ± 4.7 |
| Anthracene | 2.34 | 2.46 ± 0.1 |
| Fluoranthene | 253 | 287 ± 34 |
| Pyrene | 147 | 166 ± 21 |
| Benz[*a*]anthracene | 30.7 | 31.1 ± 3.9 |
| Chrysene | 123.5 | 123.6 ± 2.9 |
| Benzo[*b*]fluoranthene | 42.7 | 41.5 ± 2.6 |
| Benzo[*k*]fluoranthene | 18.4 | 18.95 ± 0.54 |
| Benzo[*e*]pyrene | 59.8 | 58.9 ± 2.9 |
| Benzo[*a*]pyrene | 9.27 | 9.73 ± 0.43 |
| Perylene | 6.72 | 6.8 ± 0.34 |
| Indeno[1,2,3-*c*,*d*]pyrene | 13.2 | 14.9 ± 4.5 |
| Dibenz[*a*,*h*]anthracene | — | — |
| Benzo[*g*,*h*,*i*]perylene | 22.0 | 23.7 ± 2.2 |

**Table S3**. Percentage recovery of surrogate standards in Sotalia guianensis individuals

|  | Recovery (%) | |  |  | Recovery (%) | |
| --- | --- | --- | --- | --- | --- | --- |
| Sample ID | PCB 103 | p-terphenyl-d14 |  | Sample ID | PCB 103 | p-terphenyl-d14 |
| 52476 | 95 | 92 |  | 250972 | 75 | 62 |
| 76786 | 99 | 69 |  | 212702 | 73 | 61 |
| 181676 | 72 | 61 |  | 165900 | 88 | 63 |
| 207052 | 91 | 72 |  | 167586 | 78 | 73 |
| 65420 | 73 | 73 |  | 951801 | 83 | 68 |
| 178132 | 87 | 69 |  | 48094 | 87 | 76 |
| 61722 | 81 | 97 |  | 179672 | 81 | 89 |
| 73881 | 98 | 56 |  | 60209 | 94 | 81 |
| 73981 | 84 | 76 |  | 166380 | 101 | 65 |
| 71132 | 89 | 72 |  | 32515 | 94 | 59 |
| 71886 | 87 | 90 |  | 28502 | 86 | 62 |
| 186285 | 88 | 68 |  | 100735 | 78 | 80 |
| 37604 | 78 | 53 |  | 253617 | 79 | 70 |
| 196565 | 69 | 63 |  | 17458 | 67 | 72 |
| 83099 | 71 | 81 |  | 188635 | 88 | 70 |
| 34148 | 81 | 64 |  | 32379 | 74 | 61 |
| 203823 | 86 | 58 |  | 107660 | 73 | 62 |
| 60790 | 82 | 67 |  | 23198 | 67 | 64 |
| 47084 | 102 | 62 |  | 86851 | 97 | 70 |
| 41684 | 75 | 62 |  | 32499 | 84 | 77 |
| 89779 | 76 | 79 |  | 30794 | 95 | 60 |
| 99631 | 81 | 66 |  | 244642 | 101 | 73 |
| 39024 | 78 | 57 |  | 50052 | 76 | 88 |
| 238597 | 93 | 61 |  | 157930 | 82 | 68 |
| 142667 | 81 | 55 |  | 71379 | 92 | 65 |
| 161520 | 100 | 77 |  | 60302 | 84 | 60 |
| 177908 | 103 | 67 |  | 38033 | 84 | 75 |
| 73319 | 97 | 65 |  | 35895 | 76 | 59 |
| 178636 | 82 | 66 |  | 228479 | 89 | 79 |
| 38723 | 104 | 57 |  | 18083 | 76 | 78 |
| 240518 | 72 | 61 |  | 242076 | 92 | 61 |
| 39378 | 80 | 75 |  | 758869 | 70 | 64 |
| 86353 | 70 | 69 |  | 31418 | 87 | 75 |
| 122656 | 92 | 96 |  | 832574 | 95 | 69 |
| 39004 | 80 | 73 |  | 11755 | 79 | 77 |

**Table S4**. Method detection limits (LOD) and quantification limits (LOQ) for the analyzed compounds (ng g^-1^).

| **Compounds** | **Quantification limit (LOQ)** | **Detection limit (LOD)** |
| --- | --- | --- |
| **POPs** |  |  |
| PCB 49 | 3.6 | 0.09 |
| PCB 52 | 3.6 | 0.09 |
| PCB 66 | 3.6 | 0.09 |
| PCB 77 | 3.6 | 0.09 |
| PCB 81 | 3.6 | 0.07 |
| PCB 95 | 3.6 | 0.06 |
| PCB 101 | 3.6 | 0.07 |
| PCB 110 | 3.6 | 0.06 |
| PCB 114 | 3.6 | 0.09 |
| PCB 118 | 3.6 | 0.07 |
| PCB 123 | 3.6 | 0.08 |
| PCB 138 | 3.6 | 0.05 |
| PCB 141 | 3.6 | 0.05 |
| PCB 149 | 3.6 | 0.05 |
| PCB 151 | 3.6 | 0.05 |
| PCB 153 | 3.6 | 0.12 |
| PCB 156 | 3.6 | 0.10 |
| PCB 157 | 3.6 | 0.06 |
| PCB 169 | 3.6 | 0.09 |
| PCB 174 | 3.6 | 0.06 |
| PCB 177 | 3.6 | 0.05 |
| PCB 180 | 3.6 | 0.05 |
| PCB 189 | 3.6 | 0.08 |
| PCB 194 | 3.6 | 0.07 |
| PCB 195 | 3.6 | 0.05 |
| PCB 206 | 3.6 | 0.02 |
| o,p'-DDE | 3.6 | 0.08 |
| p,p'-DDE | 3.6 | 0.08 |
| o,p'-DDD | 3.6 | 0.08 |
| p,p'-DDD | 3.6 | 0.08 |
| o,p'-DDT | 3.6 | 0.08 |
| p,p'-DDT | 3.6 | 0.08 |
| PBDE 28 | 3.6 | 0.05 |
| PBDE 47 | 3.6 | 0.05 |
| PBDE 99 | 3.6 | 0.05 |
| PBDE 100 | 3.6 | 0.10 |
| PBDE 153 | 3.6 | 0.07 |
| PBDE 154 | 3.6 | 0.06 |
| PBDE 183 | 3.6 | 0.09 |
| ɑ-HCH | 3.6 | 0.05 |
| ß-HCH | 3.6 | 0.13 |
| ƴ-HCH | 3.6 | 0.38 |
| ɗ-HCH | 3.6 | 0.02 |
| Heptachlor | 3.6 | 0.09 |
| Heptachlor Epoxide A | 3.6 | 0.09 |
| Heptachlor Epoxide B | 3.6 | 0.09 |
| Oxy-Chlordane | 3.6 | 0.13 |
| ƴ -Chlordane | 3.6 | 0.09 |
| ɑ-Chlordane | 3.6 | 0.17 |
| Endosulfan I | 3.6 | 0.04 |
| Endosulfan II | 3.6 | 0.05 |
| Mirex | 3.6 | 0.04 |
| Aldrin | 3.6 | 0.09 |
| Isodrin | 3.6 | 0.09 |
| Dieldrin | 3.6 | 0.07 |
| Endrin | 3.6 | 0.10 |
| HCB | 3.6 | 0.08 |
|  |  |  |
| **PAHs** |  |  |
| Naphthalene | 9.0 | 0.6 |
| Acenaphthylene | 9.0 | 0.1 |
| Acenaphthene | 9.0 | 0.1 |
| Fluorene | 9.0 | 0.1 |
| Dibenzothiophene | 9.0 | 0.2 |
| Phenanthrene | 9.0 | 0.3 |
| Anthracene | 9.0 | 0.1 |
| Fluoranthene | 9.0 | 0.2 |
| Pyrene | 9.0 | 0.1 |
| Benz[*a*]anthracene | 9.0 | 0.3 |
| Chrysene | 9.0 | 0.3 |
| Benzo[*b*]fluoranthene | 9.0 | 0.2 |
| Benzo[*k*]fluoranthene | 9.0 | 0.2 |
| Benzo[*e*]pyrene | 9.0 | 0.1 |
| Benzo[*a*]pyrene | 9.0 | 0.1 |
| Perylene | 9.0 | 0.1 |
| Indeno[1,2,3-*c*,*d*]pyrene | 9.0 | 0.1 |
| Dibenz[*a*,*h*]anthracene | 9.0 | 0.1 |
| Benzo[*g*,*h*,*i*]perylene | 9.0 | 0.1 |
